# The Pathology and Treatment of Trismus Nascentium

**Published:** 1846-06-01

**Authors:** 


*The Pathology and Treatment of Trismus Nascentium.* By Marion Sims, M. D. *of Montgomery, Alabama.*—American Journal of Medical Sciences, for April, 1846.

Dr. Sims suggests a novel explanation of trismus nascentium, which, however, he renders quite plausible by anatomical and physiological considerations, and sustains by facts which have fallen under the observation of himself and friends.

In the autopsy of a patient dead with this disease, he found, as the chief morbid lesion, "coagulum of blood occupying the spine in its whole length, enveloping perfectly the medulla spinalis; thicker as it approaches the brain. Spinal veins full of black blood." Billard states, that in dissections after this disease, he found "blood effused between the two laminae of the tunica aracknoidea, which filled the whole of the medullary canal from the medulla oblongata to the sacral region." Billard, strangely enough, thinks it doubtful if this be adequate to cause tetanus. If autopsies sufficiently numerous make it appear that these appearances are constant, and constitute the only uniform lesions, we should think that Dr. S. is fully authorised to assume that "its morbid anatomy consists, first in a congestion and then in a rupture of the minute veins and capillaries of the medulla spinalis."

This point established, the next question is, how is this congestion and effusion produced. Dr. Sims attributes it to the physical effects of a displacement of the occipital bone, arising frequently from the habit of mothers and nurses allowing infants to remain most of time lying on the back, and frequently with some hard substance pressing directly against the os occipitalis. We quote the following description of the rationale.

"The head by the labour is elongated in the occipito-frontal diameter to its greatest possible extent, and it is consequently diminished in its smallest dimensions, by the parietal bones overlapping the occipital through almost the whole length of the lambdoidal suture. The edge of the occipital is *always* forced up under the edge of the parietal at the posterior fontanelle *in every labor.* There is not an exception to this rule. Now, as the parietal are compressed laterally, to diminish the bi-parietal diameter, they exercise a degree of traction over the *pars occipitalis* (indepedently of the forces acting behind) drawing it upward in a line toward the fontanelle, and thus shortening the vertical diameter. After birth, the bones of the head gradually acquire their proper relative position. * * * * Now, suppose the fœtal cranium be not sufficiently ossified to regain its proper shape by its own inherent elasticity; or suppose the child is imprudently retained for a length of time in the recumbent posture on a hard mattress, or a folded blanket, with a little bit of hard, old quilt; or a bunch of dirty clothes (as we often find among negroes) wadded up and stuck under the occiput, what will be the consequence? Why, the occipital bone, instead of regaining its proper position, will be pushed further under the edge of the parietal; and the whole weight of the head resting on the occipital protuberence will thus force the *entire* "pars occipitalis" upwards. If this condition be persisted in, the whole cerebral mass will be displaced, the cerebellum will be compressed between the fossae cerebelli and the tentorium; and it will thus be tilted forwards so as to produce great pressure on the whole tract of the medulla oblongata as it rests on the basilar process of the os occipitis. What is the result of this long-continued mechanical pressure, with its peculiar displacements? The circulation through the sinuses and veins of the brain is retarded; the compression of the cerebellum obstructs the cerebellar veins; the posterior edge of the foramen magnum becomes a constricting point on that portion of the medulli-spinal veins which run forward over the anterior or lateral edges of the foramen magnum, to empty themselves into the petrosal sinuses; and the spinal venous circulation, as connected with the brain, is entirely cut off."

Dr. S. adduces six cases in which the displacement of the occipital bone was apparent, and in which change of position, so as to relieve this bone from farther pressure, was attended with a manifest amelioration of the symptoms. The latter was not successful in the cases adduced, as Dr. S. supposes, in consequence of extravasation having already taken place. The treatment based on this pathology consists in avoiding causes which promote the displacement after birth, and, if trouble have already been produced, change of position. The theory is ingenious as well as curious, and, as the writer remarks, may be readily tested by observation. There is one difficulty, however, which does not appear to have occurred to Dr. S. Why is the disease more frequent in certain localities than in others? He states that the bills of mortality at New Orleans furnish a ratio of twelve monthly. Certainly this is a vastly larger proportion than in any northern city. Is this difference explicable on the supposition that infants at the South are more generally left to lie on the back for a longer time, with a hard old quilt, or a bunch of dirty clothes tucked under the occiput!

